# Evaluation of Accelerometer-Derived Data in the Context of Cycling Cadence and Saddle Height Changes in Triathlon

**DOI:** 10.3390/s21030871

**Published:** 2021-01-28

**Authors:** Stuart A. Evans, Daniel A. James, David Rowlands, James B. Lee

**Affiliations:** 1 SABEL Labs, College of Health and Human Science, Charles Darwin University, 0810 Darwin, Australia; daniel.james@cdu.edu.au (D.A.J.); Jim.Lee@cdu.edu.au (J.B.L.); 2 School of Engineering, Griffith University, 4111 Nathan, Australia; d.rowlands@griffith.edu.au

**Keywords:** accelerometer, sensor, centre of mass, cycling, sports science

## Abstract

In the multisport of triathlon cycling is the longest of the three sequential disciplines. Triathlon bicycles differ from road bicycles with steeper seat tube angles with a change to saddle height altering the seat tube angle. This study evaluated the effectiveness of a tri axial accelerometer to determine acceleration magnitudes of the trunk in outdoor cycling in two saddle positions. Interpretation of data was evaluated based on cadence changes whilst triathletes cycled in an aerodynamic position in two saddle positions. The evaluation of accelerometer derived data within a characteristic overground setting suggests a significant reduction in mediolateral acceleration of the trunk, yielding a 25.1% decrease when saddle height was altered alongside reduced rate of perceived exertion (3.9%). Minimal differences were observed in anteroposterior and longitudinal acceleration. Evaluation of sensor data revealed a polynomial expression of the subtle changes between both saddle positions. This study shows that a triaxial accelerometer has capability to continuously measure acceleration magnitude of trunk movements during an in-the-field, varied cadence cycle protocol. Accessible and practical sensor technology could be relevant for postural considerations when exploring saddle position in dynamic settings.

## 1. Introduction

Triathlon is an endurance multisport race consisting of swimming, cycling and running over various distances. Regardless of race distance, triathletes compete for fastest overall completion time, racing each segment sequentially. Irrespective of individual disciplines, cycling takes up the most time in triathlon.

Bicycles designed for triathlon tend to differ from the ones used by road cyclists [1] as triathletes seek improvements in their aerodynamic profile by reducing their frontal projected area [2]. This is achieved by moving the saddle further forward relative to the bottom bracket compared to road cyclists [3] while placing the trunk further downwards that permit the forearms to extend onto integrated aerodynamic bars.

Saddle height is one aspect of bike setup that can affect both performance and injury [4,5]. For instance, bicycle saddle configuration dictates muscle activation [3], joint kinematics [6], and performance [4]. As saddle height modifies the mechanical work of the lower limb joints [7], alterations to saddle height will alter trunk position with performance implications. Nonetheless, triathletes often select the saddle position relative to the pedals (and therefore crank) by comfort rather than scientific knowledge. As sports medicine practitioners need to be able to advise athletes on ways to improve cycling performance, an understanding of how saddle height can influence trunk motion and the effects on performance are important.

The evaluation of kinematics in functional, sports-specific situations continues to receive attention [8]. Trunk position has been identified as an important parameter that can affect cycling performance. For instance, upper body position has been related to changes in activation of lower limb muscles [9], shown to effect cycling performance [10]. In this regard, kinematic parameters like velocity and acceleration can be used to quantify movement related to trunk position when cycling in motion. Measurement of trunk acceleration could yield more specific information about the relationship to revolutions per minute (rev/min^1^, cadence) and upper body postural movement.

Past results have reinforced that feedforward postural responses result in trunk movements with trunk orientation and centre of mass (CoM) both controlled relative to rapid limb movements [11]. Notably, phasic activation of superficial trunk muscles was consistent with that of preparatory motion with the direction of CoM motion. Similarly, Savelberg et al. [9] reported that trunk position influences EMG patterns. However, the tested positions (i.e., 20° forward and backward of the vertical) were not comparable to the aerodynamic position used by triathletes. Furthermore, magnitude of acceleration was not measured. A low level of core trunk muscle strength or stability can cause additional upper body movement. In this instance, the capacity of the lumbar-pelvic-hip complex to control lower trunk movement and preserve stability of the trunk can be compromised. Therefore, the core musculature may influence the kinematics and load-bearing capacity of the knee by determining what loads are transmitted from the trunk. In cycling, Costes et al. [12] note that an increase in power can result in intensification of acceleration forces directed to the pelvis and upper body. Taken together, poor core strength or stability combined with increases in power output could cause excessive acceleration leading to inefficient movement. The idea being that higher intensity levels are related with larger mediolateral force swaying [13] with strenuous cycling decreasing stability in the anteroposterior direction [14]. Correspondingly, an increase in workload necessitates additional upper body stabilisation [15].

A step forward in assessment would be to evaluate trunk acceleration magnitudes outside of the laboratory with easy-to-use, low cost and time efficient systems [16]. Sensors, specifically accelerometers, have been used to measure physical activity in BMX cycling [17] and to infer changes to power in indoor cycling [18]. Yet sensor technology has largely been neglected in triathlon cycling, thereby lacking quantitative data despite the possibilities to approximate trunk movements

**Figure 1:**
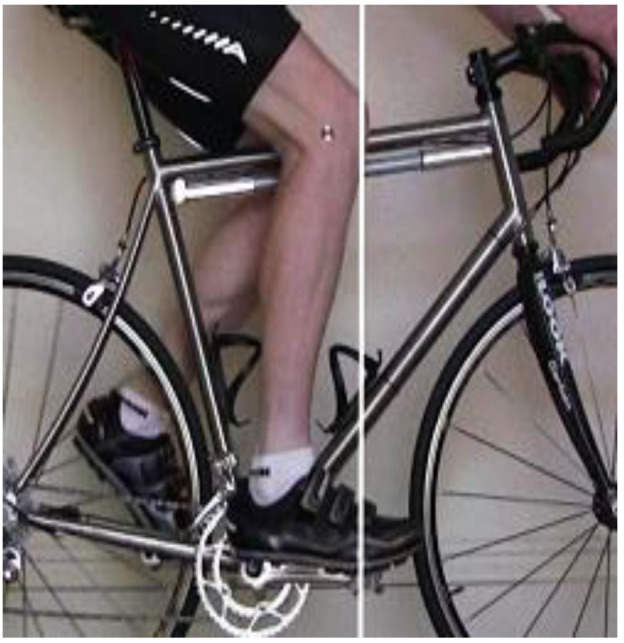
Typical KOPS approach to setting saddle height.

In the last decade the bibliography of information on saddle position has grown including static (at rest) biomechanical analysis and dynamic (while cycling) evaluations [19], with the colloquial method commonly used by triathletes referred to as the knee over pedal spindle (KOPS) technique (Figure 1). Though, some suggest this position may increase the risk of patellofemoral joint (PFJ) pain [19,20,21] or that 107% of inseam [22] or a range of 106–109% [23] is preferred. Using multiple regression, Ferrer-Roca et al. [24] explored the relationship between anthropometrics, pedalling angles, and saddle height with results supporting the correlation between saddle height, inseam length, and knee angle. From this, the authors presented an equation with 108.6–110.4% of inseam leg length proposed.

As the majority of motion and power generation occurs in the sagittal plane with compensatory movement of other body segments, unwanted or excessive movement in the frontal or transverse plane can lead to performance decreases due to increases in postural sway. The measurement of these movements is possible by accelerometers. By using sensor technology, a sports scientist or coach can monitor trunk accelerations data in real time. Therefore, quantification of acceleration magnitudes of the trunk and the determination of acceleration magnitudes paired with cadence could play an important role, providing feedback and technical indications based on sensor outputs.

A reason for the limited number of studies into trunk acceleration in triathlon cycling could be due to the difficulty of replicating kinematic analyses in a controlled laboratory environment because of differences between maxima power output performed on ergometers compared with road cycling conditions [25]. Laboratory-based monitoring and instrumentation necessitates the triathlete to remain quasi-static and in relative closeness for tethered electronic sensors [26]. The development and advances of wireless sensors has created opportunity to obtain systematic data in real-time and in the field with the capability to assess and cue changes in postural parameters. Accelerometers are unobtrusive, lightweight, inexpensive and commercially available which makes them an attractive option for field-based research [27]. Nevertheless, the mechanical robustness of the sensor is important as overground cycling conditions may change due to environmental stimuli (i.e., wind speed, terrain). To date, little attention has been given to the magnitude of trunk acceleration on the triathlon bicycle. Therefore, it is important to evaluate the effects of using wearable sensor technology in the context of overground cycling in different saddle positions.

The aim of this sensor-based approach study was to evaluate the effectiveness of a triaxial accelerometer in detecting trunk acceleration magnitude in two different saddle positions. This novel technique using sensor technology could be utilised to provide race-standard feedback on excessive accelerations of the trunk.

## 2. Materials and Methods

The purpose of this study was to evaluate the effectiveness of a sacrum-mounted sensor (accelerometer) in detecting trunk acceleration magnitudes relative to saddle positions and cadence. To accomplish this, participants cycled at varied cadence for 20 km in each saddle position whilst wearing an accelerometer. In a performance context, if one saddle position is more beneficial it will require less postural changes and therefore less acceleration magnitudes at a given cadence.

This pilot study consisted of seven recreational triathletes (age: 42 ± 11 years, height: 170 ± 6 cm, weight: 68 ± 6 kg, weekly training frequency: 7 ± 1 h, saddle height: 78 ± 0.4 cm, inseam: 75 ± 4 cm, seat tube angle (STA): 78° ± 0.49), recruited by word of mouth and social media within the local triathlon community. All participants were healthy and had no known neuromuscular or musculoskeletal disorders at the time of the study. The participants were asymptomatic of illness and free from any acute or chronic injury, as established by the American College of Sports Medicine (ACSM, 2010) participant activity readiness questionnaire (PAR-Q) [28] with a protocol approved by the University’s Research Ethics Committee (HREC 030317). Individuals used their own bicycles with integrated aerodynamic bars. Participants were asked to refrain from vigorous training 24 h prior to testing and were instructed to preserve their normal diet. All were tested using their own bicycles to eliminate the effects of unfamiliarity.

### 2.1. Methodology

A total of two outside cycling experiments were performed. Experiment 1 (day 1) was performed at the participants’ preferred/KOPS position. Experiment (day 2) was performed at an adjusted saddle height. Each experiment consisted of a 20 km overground cycle that involved 4 × 5 km (i.e., 20 km) of cycling at varied cadence. Both experiments followed the same protocol as stated in Table 1. A period of 7 days separated experiment 1 and 2.

**Figure 2:**
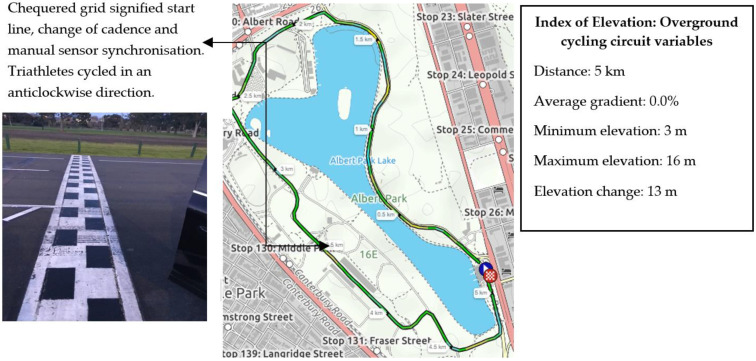
Map view and location of both experiments. The leftmost side represents the zoom view of chequered grid that signified a cadence change in both experiments. The rightmost side represents the index of elevation that contains the course variables experienced by the triathletes during both experiments. The average gradient across the 5 km circuit was 0%.

The 20 km cycle protocol for experiment 1 and experiment 2 was performed on a predominately flat circuit (wind speed 7–8 km/h, average gradient 0%) that is commonly used by triathletes for time trial (TT) performance (Figure 2). The circuit was purposely selected to avoid increased braking performance, as is common in TT performance. Furthermore, the circuit was frequently used by participants and is common within the local triathlon community due to the low technical difficulty, with little need for braking power, as is generally similar to typical courses experienced in triathlon races. As the circuit was commonly used in training and performance contexts, it therefore allowed for appropriate evaluation of the sensor relative to real-life performance application. In this sense, triathletes were able to adopt their familiar aerodynamic position (defined as elbows on the pads of the aero-handlebars with elbow angle close to 90° and the upper part of the trunk parallel to the ground) in both experiments. This position limits the need for braking given the extended trunk position and instead increases reliance on using the integrated gearing shifters located at the end of the aerodynamic bars, which differs from that used by road cyclists. Consequently, triathletes are more likely to “shift up or down” to a lower gearing ratio in order to achieve a relative cadence in order to maintain performance.

Participants were evaluated at the same time of the day (between 0700–0900), under similar environmental conditions (16–17 °C, 60–65% relative humidity). These specific times were knowingly selected due to the circuit being free from interference (i.e., vehicles). Participants commenced and completed cycling at the chequered grid whereby displacement was: Δx=xf−x0 (where xf is final position and x0 is initial position).

The chequered grid as displayed in Figure 2 signified the point where the participant changed cadence and represented the completion of the previous lap protocol and the start of the next lap protocol (i.e., 5 km). Changes to cadence were verbally communicated to participants once the front wheel of their bicycles contacted the chequered grid. To signify the completion of one 5 km lap and cadence condition, the sensor was manually synchronised by the authors as the cyclists rode past the chequered grid in order to identify synchronisation points in the raw data during post hoc analysis. Cadence was measured in revolutions per minute (rev/min^1^) with cadence ranges based on a previously established protocol [29]. Cadence was also selected due to its simplicity of measurement and that all participants had fitted cadence meters on their bicycles.

Prior to both experiments, participants performed a self-selected warm-up on their bicycles for approximately 10 min. The cadence protocol (Table 1) was the same for all participants with no additional instructions provided. Prior to performing experiment 2, a period of 15 min was permitted whereby measurements of inseam leg length were recorded using a standard tape measure in order to determine participant anthropometrics [24] and adjust saddle height. Inseam measurements were then taken and equation 1 [24] was used to estimate the adjusted saddle position with clipless pedals (i.e., 108.6–110.4% of inseam):

SH = 22.1 + (0.896*E*) − (0.15*KA*) (1)

where SH is saddle height (cm), *E* is inseam length (cm), and *KA* is the recommended knee angle (30–40°).

### 2.2. Bicycle Configuration

**Figure 3:**
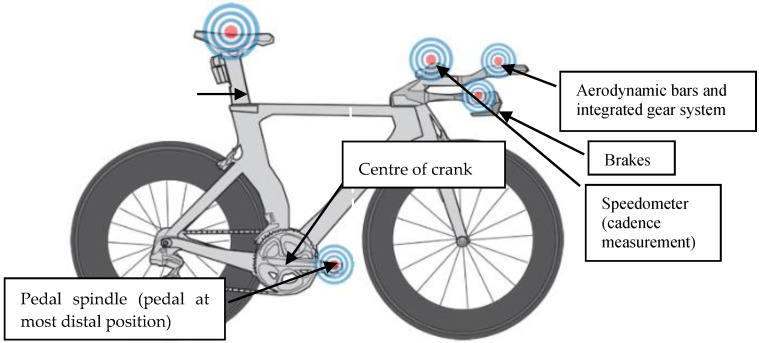
Representative diagram of key geometric measurements of triathlon bike used in study for both preferred/KOPS and adjusted saddle position. Image retrieved from bikefit.com.

Based on Equation (1), an increase in saddle height was required for all participants. In line with Gregor et al. [23] saddle height was measured from the centre of the pedal axle to the saddle top, with the pedal at the most distal end (Figure 3).

To ensure validity, measurements of knee flexion angle were manually taken by the researchers using a goniometer with participants in a static, aerodynamic position. Knee flexion was measured with the pedal placed at the bottom dead centre (180°) on the right side of the cyclist at the greater trochanter and lateral femoral condyle. Saddle position was manually adjusted according to Equation (1) before participants assumed their natural aerodynamic position and repeated the protocol from experiment one. Aside from saddle position, participants did not have bicycle configuration standardised as this would have affected muscle recruitment patterns [1].

Participants reported exertion upon completion of each 5 km lap during both experiments using the Borg 6–20 rating of perceived exertion (RPE) scale [30]. The scale is a tool for measuring an individual’s effort and exertion, breathlessness and fatigue during physical work. The scale is based on a numerical range from 6–20 where 6 means “no exertion at all” and 20 means “maximal exertion” [30]. The use of RPE and self-monitoring intensity allows participants to ensure that effort is kept within the moderate-intensity range. All participants had prior experience of using perceptual RPE scaling. Time was recorded using a Sportline 240 Econosport manual stopwatch (New York, NY, USA). Northwave tri-sonic cycling shoes (Northwave, Via Levada, Pederobba TV, Italy) with Shimano SPD-SL pedals with yellow cleats with a tolerance of approximately 6° flotation and tension were used by all participants. To standardise foot placement, the head of the first metatarsal was positioned directly above the pedal spindle with the foot placed laterally in the middle of the pedal [31]. Tight-fitting synthetic clothing was worn by all participants.

### 2.3. Instrumentation and Measurement

During measurement, an inertial measurement unit (IMU), specifically a triaxial accelerometer (52 mm × 30 mm × 12 mm, mass 23 g; resolution 16-bit, full-scale range 16 g, sampling at 100 Hz: SABEL Labs, Darwin, Australia) was fixed to participants’ spinous process (L5/S1) using double sided elastic adhesive tape. Specifically, linear accelerations at the sensor were measured on the skin over spinous processes, defined as the lumbar vertebrae position 5 (L5) and sacrum vertebrae position 1 (S1). The basis for this location is that it is the unique and closed external point to trunk movements and the point of distribution of the weighted position vectors that sum to zero.

During cycling, lower limb movement in the sagittal plane was constrained to a circular path by the geometry of the bicycle (i.e., by crank length and pedals). Within these constraints the triathlete can vary pedalling technique by changing the kinematics of their lower limbs; this change can be detected by the accelerometer. Consequently, if a triathlete has unwanted body movement when cycling (i.e., excessive mediolateral movement when the direction of travel is linear) the acceleration of that movement can be detected.

**Figure 4:**
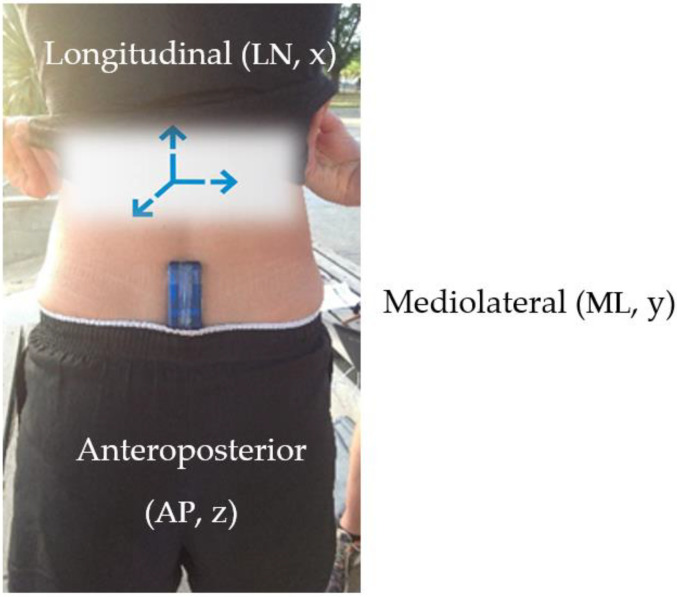
Representation of orthogonal axes orientation and sensor used in study.

For each participant and prior to commencing cycling, a static calibration was performed according to Lai et al. [32]. This process was repeated for experiment 1 (day 1) and experiment 2 (day two). This also served to check channel orientations aligned to each axis of interest [26,33]. Calibration was performed in accordance with the manufacturer’s specifications (SABEL Labs). The device hardware specifications included a ±2 g, ±4 g, ±8 g, ±16 g selectable scale. Participants were able to cycle freely overground and outside of the traditional laboratory environment due to data being stored locally on the IMU. The IMU was controlled wirelessly from the principal author via a standard Hewlett Packard PC using a comprehensive MATLAB Toolkit. This allowed for control of multiple IMUs, providing no restrictions during data capture. Data was subsequently downloaded from the IMU using a SABEL Sense software program (SABEL Sense 1.2_x64, SABEL Labs) via a CSV file. The sensor was powered by a single cell Li-Ion battery and was positioned to measure trunk acceleration data in three orthogonal planes where longitudinal (LN), mediolateral (ML) and anteroposterior (AP) aligned with X, Y and Z respectively (Figure 4).

Raw sensor data was scaled into metres per second/per second (m/s^2^), as is common in sport science literature [33,34,35]. No filtering was applied to the sensor data. As the trunk undergoes movement, the magnitude of trunk acceleration, as observed at the spinous process, will be a function of its local X, Y, and Z acceleration components. In this regard a postural change will be apparent in the local acceleration components. In this paper, trunk accelerations of each local component were compared for each participant to examine the longitudinal, mediolateral and anteroposterior changes in both saddle positions (i.e., experiment 1 and experiment 2). Accelerations were then assessed by analysing the performance of the first 5 min of each lap, excluding the initial warm up period. The purpose of this was to ensure that a steady pacing strategy and cadence stabilisation was obtained. Due to this applied methodology of the raw data, any excess braking or cornering that may have caused significant acceleration spikes would have been reduced. By assessing this epoch, the authors considered this as a ‘settling period’ and a stable baseline measurement. This also accounts for the cyclists’ anti-clockwise route around the circuit in that minimal braking or sudden cornering would have occurred given the experience of the triathletes and their familiarity of the course.

### 2.4. Statistical Analysis

The mean accelerations of trunk kinematics were then calculated for both experiments. Means and standard deviation were subsequently reported for the local X, Y and Z acceleration components along with RPE. For repeatability of measurement, the same sensor and cycle protocol was used in experiment 1 and experiment 2. Longitudinal acceleration was used to detect a change in posture and was identified where the acceleration magnitude began increasing towards its largest peak. The accelerometer was synchronised on the start and end of each lap (i.e., at the chequered grid) with data recorded continuously throughout testing before being transferred to a computer for analysis.

Data normality distribution and sphericity for each accelerometer component were evaluated by the Kolmogorov-Smirnov test with a logarithm transform applied to reduce non-uniform data distribution. A two-way ANOVA was used to test the interrelationship of cadence and acceleration magnitude on saddle position, the null hypothesis (H0) being that there is no difference to trunk acceleration magnitude between cadence and saddle position with equality between means. Coefficient of variation (CV), expressed as a percentage, was used to compare differences between trunk acceleration magnitudes in the two saddle conditions. Past studies have reported a quadratic power–cadence relationship that can be fitted with a quadratic regression [36]. Along this line, the saddle position-trunk acceleration-cadence relationship was simulated with a theoretical quadratic regression. In both positions, means were compared using a priori of 0.05.

## 3. Results

In accordance with Equation (1), adjusted saddle position was 84.4 ± 4.9 cm compared with 78 ± 0.4 cm preferred. When comparing the means of the total group (n = 7), a significant difference (*p* < 0.0001) was found in total acceleration magnitude between preferred and adjusted saddle position. There was also a significant difference found through RPE between both saddle positions (*p* < 0.0003) (Table 2).

**Figure 5:**
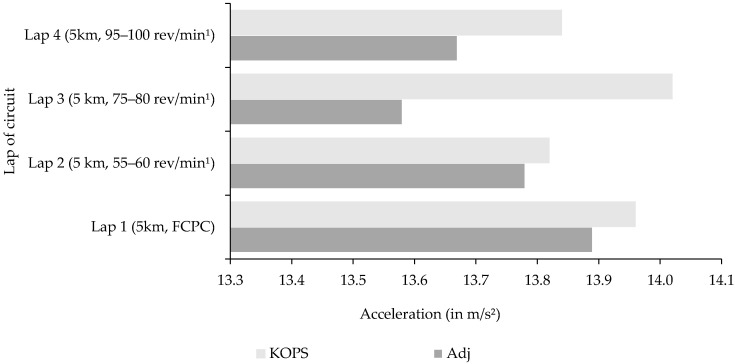
Total trunk acceleration magnitudes in two saddle positions across 4 laps of an overground circuit.

Data were separated into four subgroups that represented each completed lap of the 5 km overground circuit in order to compare differences in trunk acceleration (x, y, z). Total trunk acceleration was significantly different in the adjusted saddle position (*p* < 0.0001) in all subgroups (Figure 5). Lap 3, which was performed at 75–80 rev/min^1^, was observed to be highest in total trunk acceleration when triathletes cycled in their preferred saddle position compared to other laps. In contrast, when saddle height was adjusted triathletes recorded their lowest levels of acceleration magnitude at a cadence of 75–80 rev/min^1^.

Data were separated into four subgroups that represented each completed lap of the 5 km overground circuit in order to compare differences in trunk acceleration (x, y, z) (Table 3). The ANOVA showed significant differences between both saddle positions in mediolateral trunk acceleration in all laps and cadence conditions (*f* = 11.80, *p* < 0.001) representing a variational decrease of 25.1% in the adjusted position. The slower cadence of 55–60 rev/min^1^ was the lone increase in longitudinal acceleration in the preferred/KOPS position. In contrast, anteroposterior trunk acceleration was significantly greater in the adjusted saddle position at the same cadence (*f* = 11.90 *p* < 0.001). In addition, perceived exertion was 3.9% lower when saddle position was adjusted.

A polynomial expression was used to sum the terms containing the different exponents of variables. In this instance, the mean triaxial linear accelerations of the trunk in both the preferred/KOPS and adjusted saddle positions across the 20 km varied cadence cycle protocol was used to formulate the expression. The expression resulted in:

Preferred/KOPS saddle/adjusted saddle = 14.73pref/KOPS − 5.63adj + 0.735.(2)

where pref is preferred/KOPS and adj is adjusted.

### 3.1. Mediolateral Acceleration

**Figure 6:**
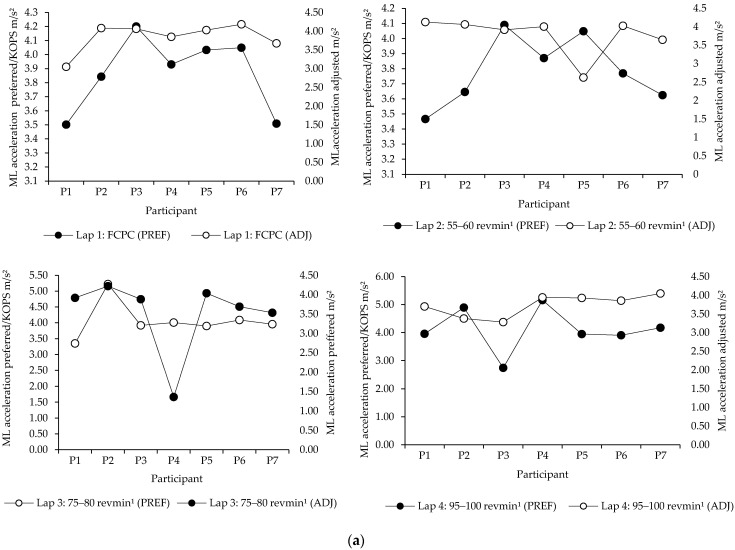

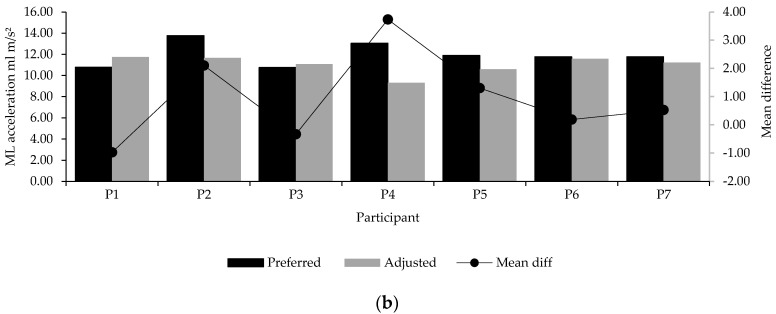
(**a**) Mediolateral trunk acceleration magnitudes per lap and cadences in two saddle positions (in m/s^2^). (**b**) Individual cumulative mediolateral trunk acceleration magnitudes across 20 km of cycling in two saddle positions. FCPC = freely chosen pedalling cadence. Acceleration presented in m/s^2^. Where PREF is preferred/KOPS saddle position.

Given that the main effects detected by the sensor were found in mediolateral trunk acceleration, within participant post hoc analysis was undertaken to examine individual responses (Figure 6). The adjusted saddle position was found to reduce total trunk mediolateral acceleration in the majority of participants. However, post hoc evaluation revealed greater variability of acceleration magnitude between participations and their respective laps. Along this line, though the total yield of reduced mediolateral trunk acceleration in the adjusted saddle height resulted in an approximate and cumulative 25.1% difference, a relative measure of variation compared to the preferred/KOPS, individual outliers (i.e., physiological capability) could have influenced performance.

### 3.2. Anteroposterior and Longitudinal Acceleration

The magnitude of anteroposterior trunk acceleration was comparable to that of longitudinal as results showed an overall yet minimal variational increase (2.3%) when saddle position was adjusted. Although small, the evaluation based on sensor data is that minimal increases in both axes occurred relative to the increased cadence and saddle height. Additionally, as triathletes cycled in an aerodynamic position in both experiments, there was no requirement to increase the vertical profile of the trunk nor trigger the sit-stand transition.

## 4. Discussion

The aim of this sensor-based approach study was to evaluate trunk accelerometer-derived data in the context of cycling cadences in two different saddle positions in triathlon. The capability of a sensor to detect changes in acceleration magnitude of the trunk in different saddle positions was coordinated with RPE scaling, with a significant reduction of perceived exertion reported when saddle position was adjusted.

Wearable technology such as a triaxial accelerometer provides an accessible and affordable tool that could support greater understanding through cycling analyses. The data used in the current study showed that a possible inverse relationship in that an adjusted saddle position resulted in reduced mediolateral acceleration magnitudes compared to the preferred/KOPS position. A possible reason for this result could be due to greater flexion trunk in triathletes when saddle height increased, symbolic of reduced cumulative mediolateral effects in the saddle. This could indicate that the preferred/KOPS methodology originally used by participants was inefficient or that the original mechanical fitting of bicycle to triathlete was incorrect. Whilst the KOPS method was used to standardise as best as possible saddle height in the participant preferred position, additional considerations variables such as crank length, stack and reach (the latter representing the vertical distance between the centre of the bottom bracket and the centre of the top of the headtube as well as the horizontal distance between the centre of the bottom bracket and the centre of the top of the headtube) could have influenced results. This suggests that the adjusted saddle height was more efficient given the 25.1% variation and acceleration difference between heights and significance throughout all laps.

Theoretical studies based on the isotonic power–velocity relationship of muscle have indicated that optimum cadence should shift to higher cadences as performance levels increase. In this regard, when participants used the preferred/KOPS method, it is feasible that greater effort was required as detected by the increased mediolateral acceleration magnitudes by the sensor, similar to that observed by Costes et al. [12]. To confirm this, there are indications that intensive cycling causes fatigue in muscles used for postural stabilisation [14]. This has led to suggestions that core strength training improves trunk stability on the saddle in order to maintain lower extremity alignment for greater effective force transmission to the pedals [37]. This advocates a need for greater upper body stabilisation when cycling, in agreement with McDaniel et al. [15] given that this extends to not only cope with the increased workload but also to balance the bike. In practical terms, using a sensor to detect and evaluate mediolateral trunk acceleration may be trainable parameter and could form a training intervention. At the same time knowledge regarding trunk acceleration magnitude and postural-trunk control in triathletes as inferred and evaluated by sensor technology is limited. A structured longitudinal strength and conditioning program with a focus on core trunk strength may lessen these effects and may be worthy for consideration of future research.

Compared with mediolateral trunk acceleration, the measurement of both longitudinal and anteroposterior trunk acceleration during the 20 km cycle displayed a minor increase when saddle height increased. Previous studies [38] showed that the neuromuscular system re-organises lower limb muscle recruitment when cyclists pedal at different saddle heights in order to sustain energy cost. Such deviations in trunk position could also lead to changes in pedal force by their effect in muscle-tendon unit lengths [39]. In this regard, it is possible that when saddle height was adjusted participants re-organised their neuromuscular system with concomitant changes in muscle-tendon unit lengths inclusive of trunk and trunk stability. This could have caused a modest acceleration in longitudinal and anteroposterior directions, particularly as cadence increased.

Whilst inferring possible relationships between trunk acceleration and RPE, muscle fibre type, pedalling cadence, oxygen consumption, ergogenic aids, biomechanical characteristics and training experience can all be listed as influencing factors. However, cadence and RPE were collected based on a previously established and accepted methodology [29] to ensure participants did not perform exhaustive activities. Whilst it was outside the scope of this study to evaluate energy cost and neuromuscular changes, additional research that combines sensors, EMG and the use of the metabolic equivalent (MET) method of caloric consumption may have merit to explore fatigue related characteristics. Whereas Galy et al. [40] required cycling to fatigue, compared to the moderately short and non-fatiguing protocol used in this pilot study, it is challenging to draw conclusions. Comparisons are further complicated since cycling in a controlled environment with measurement devices such as motion capture require participants to remain close to the device in contrast to an unobtrusive trunk-mounted accelerometer used in the present study. This warrants the need to quantify the relationship between postural stabilisation and effective force in different settings.

Similar to Kreider et al. [41], participants performed a submaximal cycling protocol to determine kinematic endpoints detected by the sensor. This represents both the uniqueness and limitation of this pilot study. Future research based on sensor technology may include: (1) evaluating the effectiveness of monitoring fatigue in overground cycling based on sensor outputs; (2) evaluation on trunk acceleration performance attributes when refinement of bicycle configuration (i.e., crank length, handlebar reach) is altered. Further evaluation may improve postural related performance characteristics. This pilot study sets the foundation for further evaluation of a sensor-based approach to trunk acceleration in triathlon cycling in order to take the measures reported here towards greater reductions in trunk accelerations.

## 5. Conclusions

The evaluation of data outputs from a triaxial accelerometer in triathlete cycling in two different saddle positions could be practically relevant. Decreased mediolateral acceleration of the trunk was detected when saddle height was adjusted with the inference that greater postural stability occurred compared to the preferred/KOPS method. Reduced mediolateral body sway has been associated with improved performance as the cyclist is able to direct greater force within the sagittal plane. An unobtrusive wireless tri-axial accelerometer with the capability to continuously measure trunk accelerations during an outdoor, varied cadence cycle could be relevant for postural considerations when exploring saddle position in dynamic settings. Whilst RPE was 3.9% lower when saddle height was adjusted, further research is warranted to assess changes in performance settings.

## Figures and Tables

**Table 1:** Cadence (in rev/min^1^) protocol performed for experiment 1 and experiment 2.

Duration (Epoch)	Lap 1	Lap 2	Lap 3	Lap 4
	5 km	5 km	5 km	5 km
Cadence	Freely Chosen pedaling cadence (FCPC)	55–60 rev/min^1^	75–80 rev/min^1^	95–100 rev/min^1^

**Table 2:** Magnitude of mean ± SD trunk acceleration (in m/s^2^) in two saddle positions in 20 km cycling.

	Preferred Saddle Position	Adjusted Saddle Position	*p* Value
Mean acceleration m/s^2^	55.64 (±0.45)	54.92 (±0.19)	<0.0001
RPE	40 (± 0.90)	38 ± (0.79)	<0.0003

**Table 3:** Descriptive data (means ± SD) from individual 5 km lap of overground cycle in two saddle positions (in m/s^2^). CV = coefficient of variation, RPE = ratings of perceived exertion, Diff = Difference between preferred/KOPS and adjusted saddle positions.

	Day 1 (Pref/KOPS Saddle Position)				Day 2 (Adjusted Saddle Position)				Difference between Means		
**Lap 1 (FCPC)**	x	y	z	RPE	x	y	z	RPE	x	y	z
Mean acc	5.09 (±0.11)	3.87 (±0.27)	5.00 (±0.17)	8.29 (±0.49)	5.12 (±0.12)	3.85 (±0.39)	4.92 (±0.19)	8.14 (±0.38)	0.59%	−0.48%	−1.59%
CV	0.02	0.07	0.03		0.02	0.1	0.04		0.20%	3.2%	0.04%
**Lap 2** (55–60 rev/min^1^)											
Mean acc	5.09 (±0.11)	3.79 (±0.23)	4.94 (±0.16)	9.57 (± 0.79)	5.05 (±0.19)	3.77 (±0.53)	4.96 (±0.2)	9.29 (±0.49)	−0.75%	−0.39%	0.51%
CV	0.02	0.06	0.03		0.04	0.14	0.04		1.50%	8.0%	0.9%
**Lap 3** (75–80 rev/min^1^)											
Mean acc	5.08 (±0.11)	4.06 (±0.57)	4.88 (±0.5)	10.71 (±1.11)	5.1 (±0.13)	3.52 (±0.14)	4.96 (±0.19)	10.11 (± 0.82)	0.27%	−13.39	1.63%
CV	0.02	0.14	0.1		0.03	0.28	0.04		0.50%	13.9%	−6.5%
**Lap 4** (95–100 rev/min^1^)											
Mean acc	5.05 (±0.1)	4.11 (±0.78)	4.68 (±0.47)	11.86 (±0.90)	5.08 (±0.11)	3.73 (±0.3)	4.86 (±0.16)	11.43 (±0.79)	0.56%	−9.20%	3.85%
CV	0.02	0.02	0.1		0.02	0.02	0.03		0.20%	0.20%	−6.7%

## Data Availability

The data presented in this study are possibly available on request. This will be subject to institutional ethical approval for release by the researchers.
